# Higher induction and maintenance infliximab trough levels are associated with radiological perianal fistula healing in pediatric patients with Crohn's disease

**DOI:** 10.3389/fped.2025.1565344

**Published:** 2025-05-16

**Authors:** Joonhyuk Son, Sungjoo Park, Seon Young Kim, Yoon Zi Kim, Hansol Kim, Wontae Kim, Sanghoon Lee, Jeong-Meen Seo, Yon Ho Choe, Mi Jin Kim

**Affiliations:** ^1^ Department of Surgery, Hanyang University College of Medicine, Seoul, Republic of Korea; ^2^ Department of Surgery, Samsung Medical Center, Sungkyunkwan University School of Medicine, Seoul, Republic of Korea; ^3^ Department of Pediatrics, Samsung Medical Center, Sungkyunkwan University School of Medicine, Seoul, Republic of Korea

**Keywords:** pediatric crohn's disease, perianal fistula, infliximab, trough level, fistula healing

## Abstract

**Background:**

Literature on the association between high infliximab (IFX) trough levels and perianal fistula response in pediatric patients with perianal fistulizing Crohn's disease (PFCD) is limited. This study aimed to evaluate the association between IFX trough levels and radiological perianal fistula healing in pediatric patients with PFCD undergoing long-term IFX treatment.

**Methods:**

The study included pediatric patients (aged <18 years) diagnosed with PFCD who received IFX treatment and underwent follow-up magnetic resonance imaging (MRI) at 1 year. The primary outcome was radiological fistula healing on MRI one year after IFX treatment.

**Results:**

A total of 82 patients were included and 57 (69.5%) achieved radiological fistula healing at the 1-year follow-up. Patients with radiological fistula healing had lower rates of reoperation (*p* = 0.021), and higher median IFX trough levels at week 2 (median, 17.6 vs. 14.1 μg/ml), week 6 (11.79 vs. 7.11 μg/ml), week 30 (3.9 vs. 1.1 μg/ml), and week 54 (7.6 vs. 3.7 μg/ml) (*p* = 0.043, 0.003, 0.007 and <0.001, respectively) compared to those who had no fistula healing. In the multivariate analysis, higher median IFX trough levels at week 6 and week 54 remained significant factors associated with radiological fistula healing (*p* = 0.039 and 0.018, respectively). Optimal cut-off IFX trough levels for radiological fistula healing showing the highest area under curve (AUC) score was 9.7 μg/ml [AUC: 0.792, 95% confidence interval (CI): 0.630–0.955; *p* = 0.005] for week 6, and 5.1 μg/ml (AUC, 0.848; 95% CI: 0.750–0.947; *p* < 0.001) for week 54.

**Conclusion:**

There was a significant association between higher serum IFX trough levels (during induction and maintenance) and radiological perianal fistula healing after 1 year of IFX treatment in pediatric patients with PFCD.

## Introduction

Perianal fistula is one of the most common manifestations in patients with Crohn's disease (CD) (1). According to the literature, the incidence of perianal fistulizing CD (PFCD) in patients with CD is between 17% and 43% (2–4). PFCD presents with various symptoms such as perianal pain, bleeding, purulent discharge, and fecal incontinence (5). Thus, achieving long-term fistula healing is crucial for patient well-being and is especially important for children and adolescents who require appropriate growth and development. However, the treatment of PFCD remains challenging and usually requires a multidisciplinary approach, including medical and surgical treatments (6). To date, surgical procedures in combination with various immunomodulator therapies, such as monoclonal antibodies against tumor necrosis factor (TNF), have shown the best outcomes in terms of fistula healing with fewer side effects (7–9).

Infliximab (IFX), the most widely used anti-TNF agent for PFCD, has shown effectiveness in the treatment of perianal fistulas and has been recommended as first-line therapy for PFCD (10). In recent years, there has been growing evidence of an association between IFX trough levels and perianal fistula response, and few clinicians have assessed the optimal trough level required to achieve fistula healing (11–15). These existing studies mainly set early fistula response as the primary endpoint. Unfortunately, there is still no consensus on the endpoint for IFX treatment, resulting in a lack of standardized guidelines for long-term treatment plans for patients with PFCD undergoing IFX treatment. Notably, these studies have been based on adult patients, and to our knowledge, only one study with a small sample size has focused on pediatric patients (16). A recent multicenter randomized controlled trial (RCT) highlighted the importance of radiological fistula healing for achieving long-term remission in PFCDs (17). Given these circumstances, our aim was to analyze the association between IFX trough levels and radiological fistula healing in pediatric patients with PFCD undergoing long-term IFX treatment.

## Materials and methods

### Study design and patient selection

**Figure 1:**
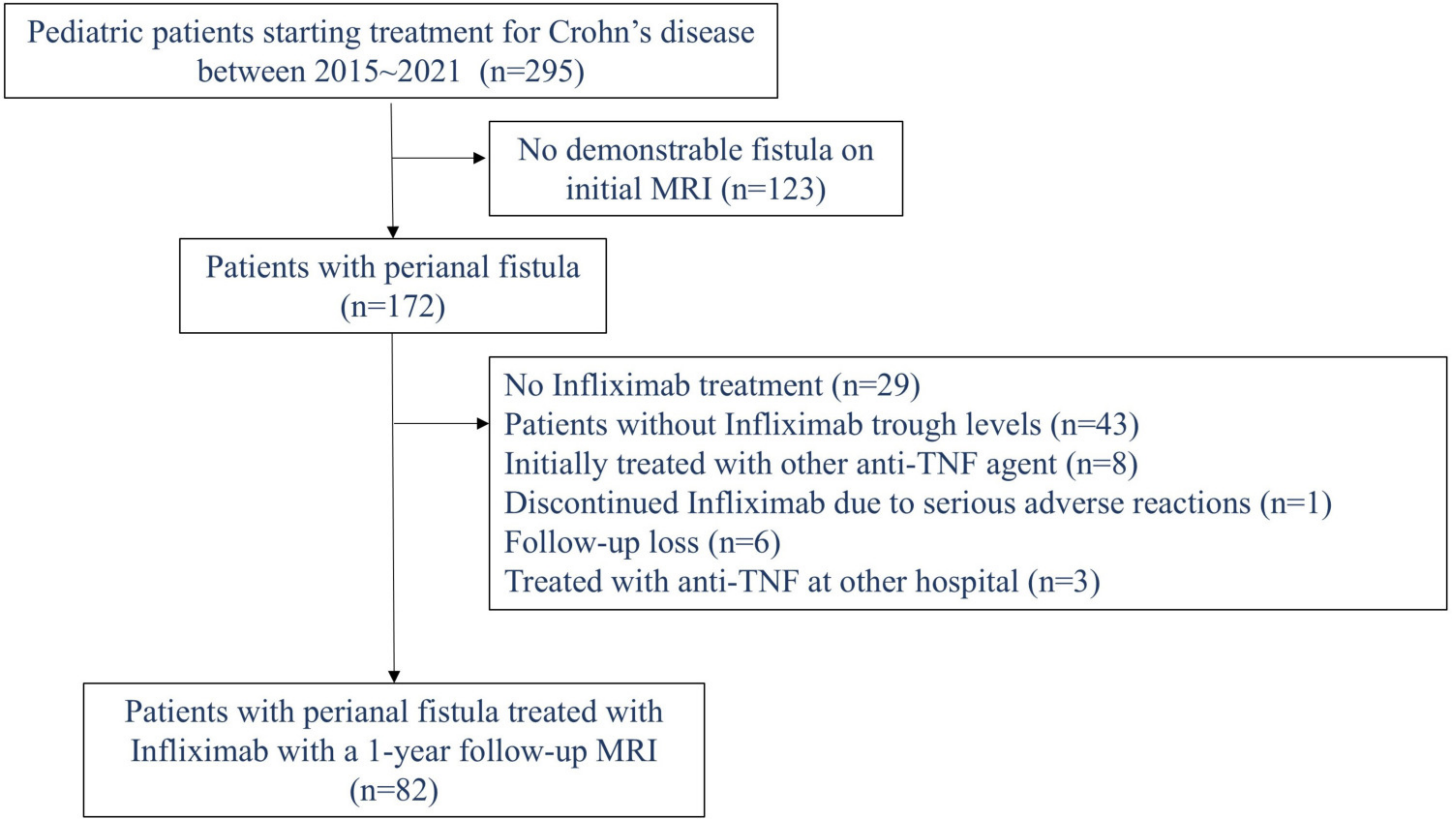
Flowchart of patient selection (inclusion and exclusion criteria) in pediatric patients with Crohn’s disease.

This single-center retrospective study was conducted at the Samsung Medical Center (Seoul, Republic of Korea). We reviewed the medical records of all patients under 18 years of age who were newly diagnosed with CD between January 2015 and December 2021. Only patients with a demonstrable perianal fistula on their initial magnetic resonance imaging (MRI) at diagnosis, who were treated with IFX, and had a 1-year follow-up MRI were included. Those who developed a perianal fistula after diagnosis were excluded. Additional exclusion criteria included: (1) no history of IFX treatment, (2) lack of serum IFX trough level data, (3) initial treatment with a different anti-TNF agent, (4) primary non-responders to IFX therapy or discontinuation before reaching maintenance therapy due to severe adverse events, and (5) absence of a 1-year follow-up MRI. Figure 1 illustrates the patient inclusion process.

Patients who required a switch to another anti-TNF agent due to adverse events or secondary loss of response (LOR) were classified as non-responders in the analysis. At our center, patients are switched to another therapy only if they experience severe adverse effects or significant symptom worsening, as reverting to the previous medication is nearly impossible due to insurance restrictions. Consequently, switches are rarely made at our institution.

At our center, a step-up approach remains standard practice based on local reimbursement criteria, allowing biological therapy (anti-TNF therapy) only after two months of failure with corticosteroids, exclusive enteral nutrition, and conventional immunomodulators. Despite the lack of clear evidence supporting the use of 5-aminosalicylates in patients with CD according to current guidelines, they are still routinely initiated in all pediatric patients with CD.

IFX was administered intravenously at a dose of 5 mg/kg at weeks 0, 2, and 6 of the induction period, followed by maintenance therapy every eight weeks. All patients received standard proactive drug monitoring during induction and maintenance treatment. However, since trough level testing is not covered by insurance and is relatively expensive, some parents choose to decline the test. As a result, trough levels were measured only when parental consent was provided. Otherwise, testing was primarily performed when LOR was suspected. Consequently, among the patients who were treated with IFX, approximately 65% of the patients underwent proactive drug monitoring.

Serum IFX trough and anti-infliximab antibody (ATI) levels were measured immediately before the scheduled IFX infusion. The trough levels measured at weeks 2 and 6 were included to analyze drug concentrations during the induction period, and the trough level at week 14 (before the 4th dose of IFX) was also included as a variable to analyze the post-induction period. The trough levels at weeks 30 (6 months) and 54 (1 year) were included as variables to check drug concentrations during the maintenance period. The trough level of IFX was measured using an IDK monitor® IFX drug-level enzyme-linked immunosorbent assay (ELISA) kit (Immundiagnostik AG, Bensheim, Germany). For ATIs, the IDK monitor® IFX free ADA ELISA (Immundiagnostik AG, Bensheim, Germany) kit was used. The trough levels at weeks 2 and 6 were measured for reference to assess the patient's response to the medication. Active dose adaptation was initiated from week 14 onward, targeting a therapeutic window of 5 µg/ml during maintenance therapy (18). Surgical treatment was performed by a professional pediatric surgeon when patients had significant perianal symptoms, including pain and purulent discharge, with a visible fistula opening.

Patients typically undergo an initial MRI examination during the diagnostic process, and all patients receive this initial examination before starting treatment with IFX. A follow-up MRI is then performed one year after initiating IFX treatment, with subsequent MRIs scheduled every two years. All MRI scans were interpreted by three experienced radiologists specializing in pediatric radiology, using a standardized and unified description format.

This study was approved by the Institutional Review Board (IRB) at Samsung Medical Center (IRB File No. 2023-11-133). This is an IRB-approved retrospective study, all patient information was de-identified and the requirement for informed consent was waived owing to the retrospective nature of the study. The study was performed in accordance with the ethical guidelines of the 1964 Declaration of Helsinki.

### Data collection and definition

Baseline demographics, including sex, age, body mass index, Pediatric Crohn's Disease Activity Index (PCDAI) score, white blood cell (WBC) count, erythrocyte sedimentation rate (ESR), C-reactive protein (CRP) level, and serum albumin level, were collected. Disease location and behavior at diagnosis was assessed according to the Paris classification (19). The severity of mucosal involvement was assessed using the Simple Endoscopic Score for Crohn's disease (SES-CD). Perianal fistulas were classified according to Park's classification (20); superficial, intersphincteric, transsphincteric, suprasphincteric, or extrasphincteric. On the basis of the American Gastroenterological Association (AGA) classification, patients with superficial, low intersphincteric, or low transsphincteric fistulas were classified into the simple fistula group. Patients with high fistulas (high intersphincteric, high transsphincteric, extrasphincteric, or suprasphincteric), multiple external openings, concomitant perianal abscesses, rectovaginal fistulas, or anorectal strictures were classified into the complex fistula group (21).

The primary outcome was radiological fistula healing achieved at the 1-year follow-up MRI exam. We adapted the definition of radiological fistula healing from previous literature (PISA-II trial) (22): a completely hypointense tract on T2-weighted sequences regardless of fat suppression, and no enhancement after contrast administration on a fat-suppressed T1-weighted sequence. The secondary outcome was radiological fistula recurrence, which was defined as when a recurrent fistula was detected at the second or third MRI follow-up among patients who achieved radiological fistula healing at the 1-year MRI follow-up.

### Statistical analysis

For continuous variables, the Mann–Whitney *U* test was used. The chi-squared test or Fisher's exact test was used to compare categorical variables. Comparative data for continuous variables are presented as median values with interquartile ranges (IQR). Logistic regression analysis was performed to determine the predictive factors for radiological fistula healing. Variables with *P*-values of <0.05 were included in the multivariate analysis. The results are expressed as adjusted odds ratios (ORs) with 95% confidence intervals (CIs). Statistical significance was set at *P*-values of <0.05. Receiver operating characteristic (ROC) curve analysis was conducted to evaluate the accuracy of the optimal cut-off value of IFX trough levels for predicting radiological fistula healing.

## Results

### Patient characteristics

This study included 82 pediatric patients with PFCD who initiated IFX therapy and underwent a follow-up MRI at 1 year. The median age at diagnosis was 14.2 years (IQR: 12.7–16.4 years). According to Park's classification, the most common perianal fistula was the intersphincteric type (51 patients, 62.2%), followed by the transsphincteric (30.5%), extrasphincteric (6.1%), and superficial (1.2%) perianal fistulas. None of the patients had suprasphincteric fistulas. A total of 31 patients (37.8%) had two or more fistulas on initial MRI examination, 52 patients (63.4%) were categorized as having complex fistulas, and 46 patients had a concomitant perianal abscess (56.1%) (Table 1).

**Table 1:** Baseline characteristics of the patients.

Variables	*n* = 82
Male sex, *n* (%)	74 (90.2)
Age (year) at diagnosis, median [IQR]	14.2 [12.7–16.4]
Body mass index (kg/m^2^), median [IQR]	19.5 [17.4–22.1]
Disease location, *n* (%)	
Ileal	27 (32.9)
Ileocolonic	55 (67.1)
Disease behavior, *n* (%)	
Non-stricturing non-penetrating	68 (82.9)
Stricturing	13 (15.9)
Penetrating	1 (1.2)
Perianal fistula subtype, *n* (%)	
Superficial	1 (1.2)
Intersphincteric	51 (62.2)
Transsphincteric	25 (30.5)
Extrasphincteric	5 (6.1)
Complex perianal fistula, *n* (%)	52 (63.4)
Number of fistulas, *n* (%)	
1	51 (62.2)
2	24 (39.3)
>3	7 (8.5)
Perianal abscess, *n* (%)	46 (56.1)
SES-CD score at diagnosis, median [IQR]	7 [3–10]
PCDAI score at initial presentation, median [IQR]	30 [20.0–37.5]
WBC (/µl) at diagnosis, median [IQR]	8,700 [6,570–10,630]
ESR (mm/hr) at diagnosis, median [IQR]	38.0 [16.8–64.5]
CRP (mg/dl) at diagnosis, median [IQR]	1.60 [0.39–2.84]
Albumin (g/dl) at diagnosis, median [IQR]	4.0 [3.7–4.4]

IQR, interquartile range; SES-CD, simple endoscopic score for crohn’s disease; PCDAI, Pediatric Crohn’s Disease Activity Index.

A total of 51 patients (62.2%) underwent surgical intervention for the treatment of perianal fistulas. Among them, nine (11.0%) required reoperation for perianal disease within a year. Seton drainage (52.9%) was the most commonly performed surgical procedure. The median time from diagnosis to initiation of IFX therapy was 67 days (IQR: 45–88 days). At the time of the 1-year follow-up, 57 patients (69.5%) achieved radiological fistula healing (Table 2).

**Table 2:** Clinical outcomes of the patients.

Variables	*n* = 82
Anal surgery, *n* (%), (*n* = 51)	
Incision and drainage only	12 (23.5)
Seton procedure with or without abscess drainage	27 (52.9)
Fistulotomy	3 (5.9)
Fistulectomy	9 (17.6)
Concomitant antibiotics, *n* (%)	42 (51.2)
Initial treatment with 5-ASA and AZP, *n* (%)	79 (96.3)
Concomitant corticosteroids, *n* (%)	9 (11.0)
IFX Trough level at week 2 (μg/ml), median (IQR)	16.74 [13.03–20.62]
IFX Trough level at week 6 (μg/ml), median (IQR)	9.78 [6.98–15.96]
IFX Trough level at week 14 (μg/ml), median (IQR)	3.26 [1.70–5.68]
IFX Trough level at week 30 (μg/ml), median (IQR)	3.48 [1.57–4.98]
IFX Trough level at week 54 (μg/ml), median (IQR)	5.58 [3.72–9.50]
Median time from first anal symptom to IFX therapy, days [IQR]	146 [90–316]
Median time from diagnosis to IFX therapy, days [IQR]	67 [45–88]
Presence of ATI, *n* (%)	18 (22.0)
Dose intensification, *n* (%)	19 (23.2)
Switched to another anti-TNF agent, *n* (%)	3 (3.7)
Reoperation, *n* (%)	9 (11.0)
Radiological fistula healing at 1 year follow-up, *n* (%)	57 (69.5)

5-ASA, mesalazine; AZP, azathioprine; IFX, infliximab; IQR, interquartile range; ATI, anti-infliximab antibody; TNF, tumor necrosis factor.

### Clinical factors associated with radiological fistula healing

**Figure 2:**
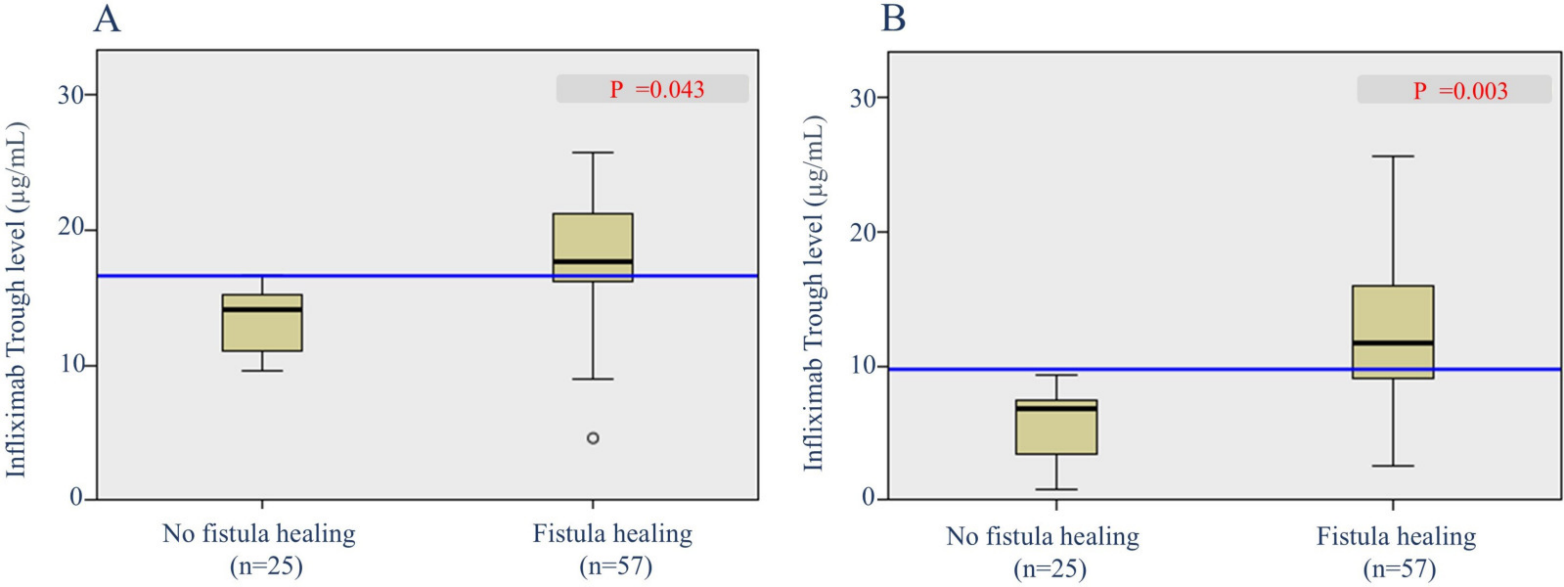
Box plots of infliximab trough levels during induction in patients with and without radiological fistula healing. **(A)** Trough levels at week 2 and **(B)** trough levels at week 6.

**Figure 3:**
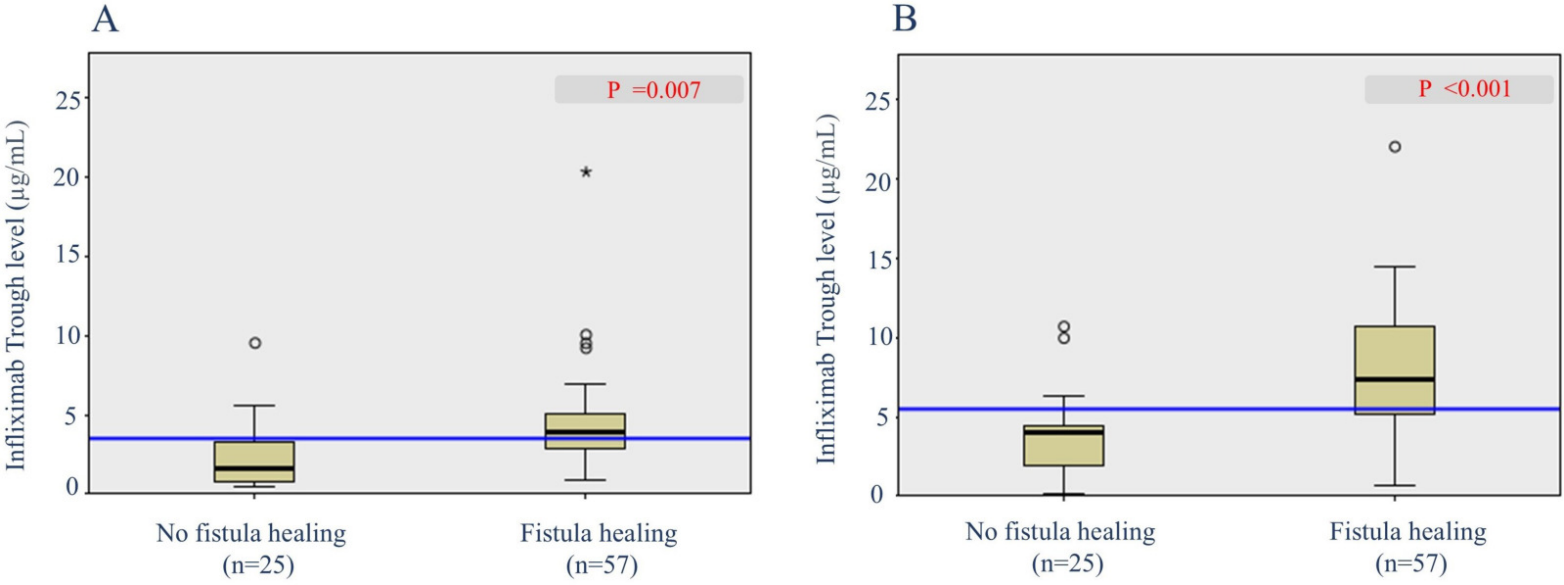
Box plots of infliximab trough levels during maintenance in patients with and without radiological fistula healing. **(A)** Trough levels at week 30 and **(B)** trough levels at week 54.

To determine the predictive factors associated with radiological fistula healing, a comparative analysis was performed (Table 3). Most characteristics, including initial laboratory results, PCDAI score, disease phenotypes, SES-CD score, perianal fistula subtypes, and treatment methods, were not statistically different between the two groups. However, patients with radiological fistula healing had significantly lower rates of reoperation (*p* = 0.021) and higher median IFX trough levels at week 2 [median, 17.6 vs. (vs.) 14.1 μg/ml], week 6 (median, 11.79 vs. 7.11 μg/ml), week 30 (median, 3.9 vs. 1.1 μg/ml), and week 54 (median, 7.6 vs. 3.7 μg/ml) (*p* = 0.043, 0.003, 0.007 and <0.001, respectively) compared to those without fistula healing (Figures 2, 3). According to the multivariate logistic regression analysis (Table 4), higher median IFX trough levels at week 6 (OR, 1.212; 95% CI, 1.009–1.456) and week 54 (OR, 1.460; 95% CI, 1.068–1.996) remained significant factors associated with radiological fistula healing (*p* = 0.039 and 0.018, respectively).

**Table 3:** Comparative analysis of factors associated with radiological fistula healing.

Variables	Fistula healing (*n* = 57)	No fistula healing (*n* = 25)	*P*-value
Baseline factors			
Age at diagnosis, median [IQR]	14.3 [12.9–16.5]	13.9 [12.4–15.6]	0.462
Body weight at diagnosis, median [IQR]	50.5 [43.7–61.4]	54.1 [43.3–60.5]	0.526
L1 location at diagnosis, *n* (%)	17 (29.8)	10 (40.0)	0.367
B1 behavior at diagnosis, *n* (%)	47 (82.5)	21 (84.0)	0.569
Complex fistula, *n* (%)	34 (59.6)	18 (72.0)	0.285
Multiple fistulas ≥2, *n* (%)	19 (33.3)	12 (48.0)	0.207
Perianal abscess, *n* (%)	31 (54.4)	15 (60.0)	0.637
SES-CD score at diagnosis, median [IQR]	6 [3–9]	10 [4–12]	0.098
PCDAI score, median [IQR]	30 [20.0–37.5]	30 [17.5–38.7]	0.786
WBC (/µl), median [IQR]	8,700 [6,650–10,800]	8,380 [5,950–10,050]	0.516
ESR (mm/hr), median [IQR]	38.0 [18.5–66.0]	37.0 [10.0–45.5]	0.323
CRP (mg/dl), median [IQR]	1.56 [0.46–3.03]	1.93 [0.32–2.51]	0.497
Albumin (g/dl), median [IQR]	4.0 [3.7–4.4]	4.2 [3.7–4.4]	0.770
Treatment factors			
Anal surgery, *n* (%)	35 (61.4)	16 (66.7)	0.654
Use of antibiotics, *n* (%)	26 (45.6)	16 (64.0)	0.125
Use of corticosteroids, *n* (%)	7 (12.3)	2 (8.0)	0.715
Reoperation, *n* (%)	3 (5.3)	6 (24.0)	0.021
Time from first symptom to IFX (days), median (IQR)	143 [97–319]	159 [79–317]	0.755
Time from diagnosis to IFX (days), median (IQR)	67 [45–96]	82 [43–88]	0.884
IFX Trough level at week 2 (μg/ml), median (IQR)	17.6 [15.4–21.5]	14.1 [10.5–16.5]	0.043
IFX Trough level at week 6 (μg/ml), median (IQR)	11.79 [8.18–17.6]	7.11 [2.10–8.11]	0.003
IFX Trough level at week 14 (μg/ml), median (IQR)	3.62 [2.1–5.8]	1.4 [1.0–5.1]	0.077
IFX Trough level at week 30 (μg/ml), median (IQR)	3.9 [2.8–5.1]	1.1 [0.5–2.3]	0.007
IFX Trough level at week 54 (μg/ml), median (IQR)	7.6 [5.2–11.3]	3.7 [1.5–4.3]	<0.001
Presence of ATI, *n* (%)	11 (19.3)	7 (28.0)	0.381
ATIs concentration (AU/ml), median [IQR], (*n* = 18)	40.5 [28.4–50.25]	45.1 [23.8–63.7]	0.258
Dose intensification, *n* (%)	13 (22.8)	6 (24.0)	0.906

IQR, interquartile range; L1, ileal disease; B1, non-stricturing non-penetrating; SES-CD, simple endoscopic score for crohn's disease; PCDAI, Pediatric Crohn's Disease Activity Index; IFX, infliximab; ATI, anti-infliximab antibody.

**Table 4:** Logistic regression analysis of factors associated with radiological fistula healing.

Variables	Univariate logistic regression		Multivariate logistic regression	
	Adjusted OR (95% CI)	*P* value	Adjusted OR (95% CI)	*P* value
Reoperation	0.176 [0.040–0.774]	0.021	0.209 [0.014–3.088]	0.254
IFX Trough level at week 2	1.081 [0.920–1.271]	0.343		
IFX Trough level at week 6	1.214 [1.027–1.434]	0.023	1.212 [1.009–1.456]	0.039
IFX Trough level at week 30	1.497 [1.001–2.240]	0.05		
IFX Trough level at week 54	1.595 [1.221–2.083]	0.001	1.460 [1.068–1.996]	0.018

OR, odds ratio; IFX, infliximab.

### Optimal IFX trough level cut-off required to achieve radiological fistula healing

**Figure 4:**
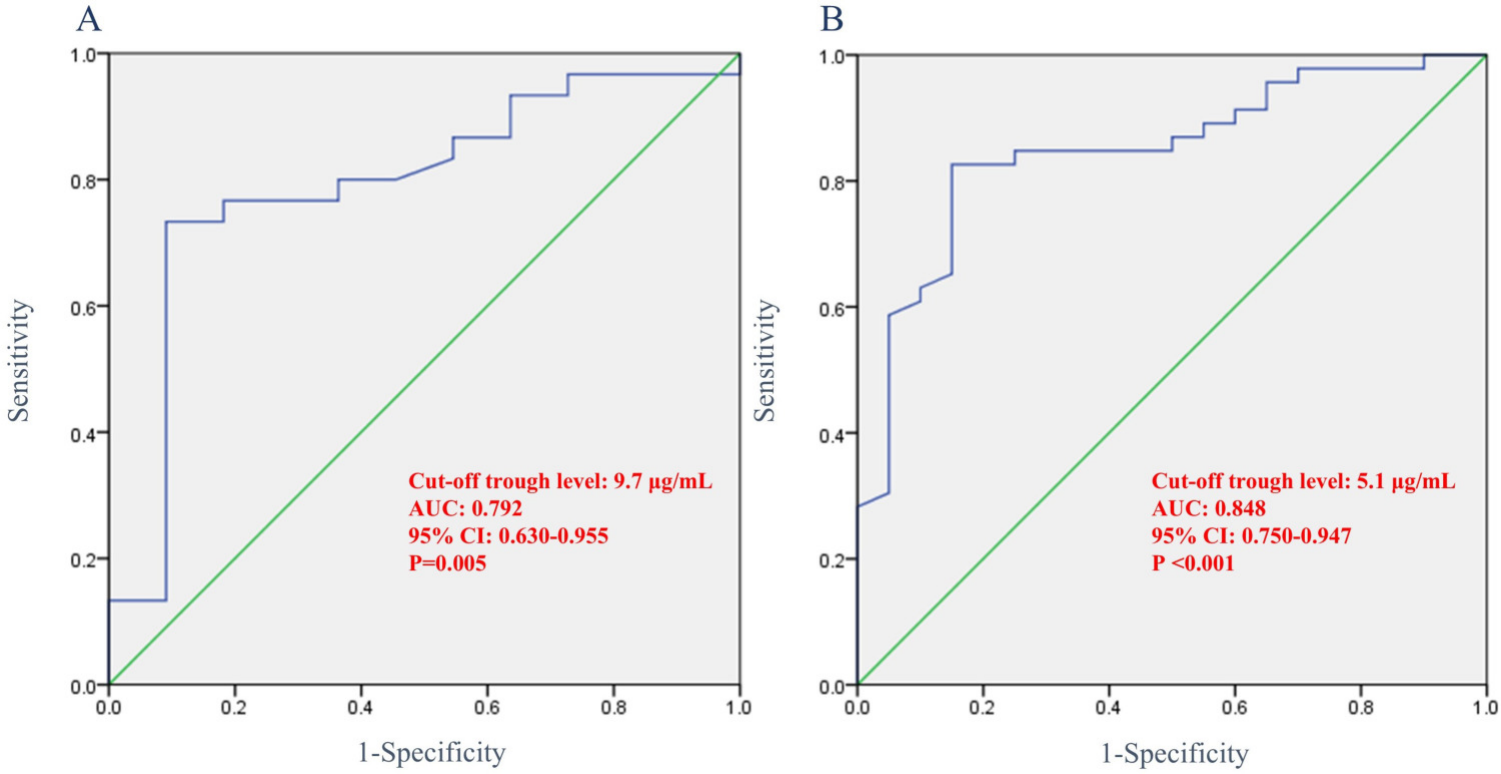
Receiver operating characteristic curve showing the association between infliximab trough levels and radiological fistula healing. **(A)** Trough levels at week 6 and **(B)** trough levels at week 54. AUC, area under curve; CI, confidence interval.

ROC analysis revealed that the optimal IFX cut-off trough level at week 6 needed to achieve radiological fistula healing was 9.7 μg/ml (AUC, 0.792; 95% CI: 0.630–0.955; sensitivity, 70.0%; specificity, 90.9%; *p* = 0.005). The optimal cut-off trough level at week 54 was 5.1 μg/ml (AUC, 0.848; 95% CI: 0.750–0.947; sensitivity, 78.2%; specificity, 85.0%; *p* < 0.001) (Figure 4).

### Analysis of patients who switched to another anti-TNF agent

**Figure 5:**
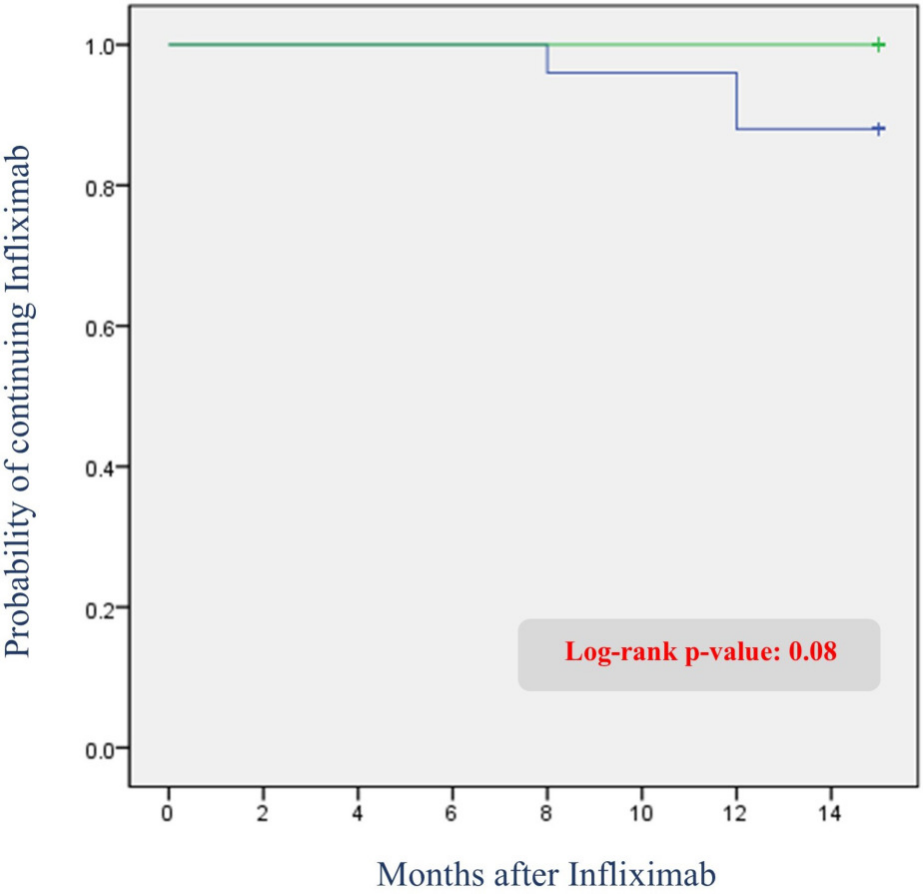
Kaplan–meier curve of infliximab discontinuation due to adverse events or secondary loss of response: comparison between patients with and without radiological fistula recurrence.

Among all patients, three (3.7%) switched from IFX to another anti-TNF agent within one year. One patient switched at week 30 due to persistently low trough levels (0.48 μg/ml) and severe worsening of perianal symptoms despite dose intensification. The remaining two patients switched at week 54; although neither experienced significant symptom worsening, both had persistently low trough levels (0.7 and <0.1 μg/ml). Consequently, dose intensification was initially planned for both patients at the one-year mark. However, one of these patients showed disease progression on the one-year MRI and colonoscopy, prompting a direct switch to an alternative anti-TNF agent without dose intensification. The other patient similarly showed no symptom aggravation but developed mild adverse effects, including urticaria starting after the week 30 dose and mild dyspnea at week 54. As a result, no dose intensification was attempted, and the medication was switched immediately after the week 54 dose. The Kaplan–Meier survival curve for infliximab discontinuation is shown in Figure 5.

### IFX trough level and radiological fistula recurrence

**Figure 6:**
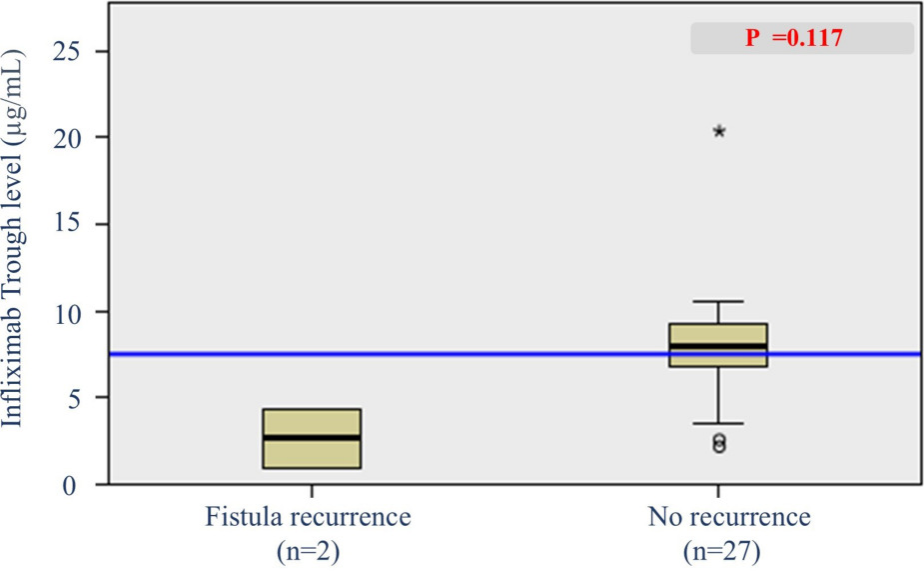
Box plot of infliximab trough levels in patients with and without radiological fistula recurrence.

Of the 57 patients who achieved radiological fistula healing at 1 year, 29 patients underwent second or more follow-up MRIs. Analysis of radiological fistula recurrence was performed in these 29 patients. Among them, 2 patients (6.9%) showed fistula recurrence on MRI. The patients with radiological fistula recurrence showed a lower trough IFX level measured within 8 weeks of MRI than those with no fistula recurrence [median, 7.90 [6.21–9.46] vs. 2.625 [0.92–4.33] μg/ml] (Figure 6). However, this difference was not statistically significant (*p* = 0.117).

## Discussion

To the best of our knowledge, this study was conducted with the largest number of pediatric patients with PFCD to investigate the association of IFX trough levels and perianal fistula healing. Herein, we present a significant association between IFX trough levels, both induction and maintenance, and radiological perianal fistula healing at the 1-year follow-up. This highlights the importance of proactive therapeutic drug monitoring in optimizing treatment outcomes for patients with PFCD.

PFCD is a disease that causes serious complications, and long-term management of the fistula remains challenging for clinicians. The 2004 ACCENT-II trial reported efficacy and feasibility of IFX in the management of PFCD (23) and a worldwide consensus on treatment guidelines has been established since then. Moreover, through various studies and consensus meetings, the use of IFX for PFCD has been recommended in the pediatric population (6). According to the results of the PISA-II RCT trial conducted in 2022 (22), fistula healing in the adult population was more frequent in the combination therapy group treated with anti-TNF and surgical treatment than in the single therapy group treated with anti-TNF treatment alone. According to a systematic review by Forsdick et al. (7), a combination of medical and surgical treatment is also necessary for pediatric patients with PFCD. These results emphasize the importance of a multidisciplinary approach involving both surgeons and pediatricians for the optimal treatment of PFCD. Although IFX and other anti-TNF agents have brought about significant progress in the management of PFCD in the past decades, there are still issues that need to be overcome, such as identifying optimal therapeutic trough levels, achieving long-term fistula healing without recurrence, and determining the appropriate timing for IFX discontinuation.

Numerous studies have investigated the association between the trough levels of anti-TNF agents and treatment response in patients with CD. Despite the various clinical endpoints in luminal disease, the AGA guidelines recommend a cut-off level of 5.0 μg/ml as a therapeutic target to achieve mucosal healing (24). In the pediatric population, several studies have explored the relationship between mucosal healing and the therapeutic window (25–27). However, to date, there are no established guidelines specifying optimal IFX trough levels for mucosal healing in pediatric patients with CD. Regarding PFCD, no therapeutic window has been proposed in either adult or pediatric populations. Although some studies have reported an association between higher IFX trough levels and fistula healing in adults (11–15, 28), to date, only one study has examined this relationship in pediatric patients with PFCD (16). We reviewed these studies and summarized the study design and cut-off trough levels for each study in Table 5. We found that the definition of primary outcomes differed among the studies. Some studies set the cessation of drainage and the absence of an external opening as the primary outcomes, whereas only a few institutes define fistula healing according to MRI results. Additionally, the timing of serum IFX trough level measurements differed between the institutes. These varied study designs among institutions make it difficult to reach a consensus on the optimal cut-off trough levels to achieve fistula healing. To provide a standard therapeutic cut-off trough level of anti-TNF agents for perianal fistula management, we suggest that all institutions should unify the primary endpoint and definition for fistula healing.

**Table 5:** Previous literature describing the association between IFX trough levels and fistula response in PFCD.

Study, year	Country	Patient number	Primary outcome and definition	Trough level* (μg/ml)	Timing of trough level measurement
Davidov et al. (11) 2017	Israel	36	Fistula response (decrease or cessation in fistula drainage) at week 14	9.25	Cut-off at week 2
				7.25	Cut-off at week 6
Yarur et al. (15) 2017	USA	117	Fistula healing (absence of drainage) at the time of endoscopic exam	10.1	Cut off at maintenance (Trough level measured within 4 weeks of an endoscopic and perianal exam)
			Fistula closure (absence of skin opening, and mucosal healing)	10.1	
El-Matary et al. (16) 2019	Canada	52	Fistula healing (decrease or cessation of fistula drainage, closure of a previously identified fistula opening) at week 24	12.7	Cut-off at week 14
Strik et al. (14) 2019	Netherland	47	Fistula closure (absence of purulent discharge and/or fistula closure on MRI)	5.0	Cut-off (No description on timing of trough level measurement)
Plevris et al. (13) 2020	UK	29	Fistula healing (spontaneous discharge or no discharge on palpation in the absence of seton drainage)	7.1	Cut off at maintenance (Trough level measured within ±4 weeks of a perianal fistula assessment)
			Fistula closure (absence of an external skin opening)	7.1	
Gu et al. (12) 2022	Australia	66	Fistula healing (cessation of fistula drainage, with or without a seton)	6.1	Cut-off at maintenance (within ±12 weeks of assessment)
Papamichael et al. (28) 2021	Multiple western countries	282	Complete fistula response (absence of draining fistulas) at week 14	15/6.1	Cut-off at week 6/week 14
		161	Composite remission (Complete fistula response with CRP normalization) at week 14	15/7.2	

IFX, infliximab; PFCD, perianal fistulizing Crohn's disease.

*Values indicate the cut-off infliximab trough levels associated with achieving the primary outcome in each study.

Notably, in patients with PFCD, therapeutic drug monitoring should aim to achieve remission in both luminal and perianal symptoms. This necessitates adjusting the target trough level based on a combination of endoscopic findings, MRI results, and subjective symptom improvement. Future research should comprehensively analyze trough levels in relation to various endpoints, including mucosal healing (or transmural healing) and fistula healing, to ultimately develop clear and evidence-based guidelines for IFX trough level targets in patients with PFCD.

Another important consideration regarding the therapeutic window for pediatric patients with CD is the increased likelihood of underdosing in younger children. Evidence suggests that pediatric patients under the age of 10 are at greater risk of being underdosed (26). Additionally, van Hoeve K et al. reported that patients weighing less than 30 kg are more prone to underdosing and may require higher trough levels to achieve optimal outcomes (29). In our study, there were only two patients with a body weight of less than 30 kg, and only one of them achieved fistula healing. Similarly, our cohort included only four patients under the age of 10, and their trough levels did not significantly differ from those of patients aged 10 years or older at any time point. These findings may be attributed to the small sample size in these groups, limiting our ability to detect a statistically significant result. Further studies with larger cohorts are warranted to clarify this issue.

Upon reviewing the literature assessing optimal IFX trough levels, we found that most studies focused on the early response or failure of IFX therapy, and little information exists on long-term outcomes and late recurrences (Table 5). A *post-hoc* analysis of the PISA-II trial by Meima et al. found that fistulas recurred only in patients who did not achieve radiologic healing within 18 months, with a median follow-up of 5.7 years (17). Based on these results, the authors proposed that radiological fistula healing should be the therapeutic target for PFCD treatments. In our study, two patients showed slightly high signal intensity in the fistula tract at the second follow-up MRI, which we defined as radiological fistula recurrence. However, both patients had no recurrence of perianal symptoms, such as discharge or anal pain, implying that clinical remission was maintained until the last follow-up. Therefore, we support the idea of authors suggesting that the ultimate treatment goal for the management of PFCD should be radiological healing of the fistula rather than clinical fistula closure to ensure long-term healing. We also believe it is important to continue IFX even if clinical closure is achieved and to continuously check serum trough levels until radiological healing is confirmed.

Another critical issue in managing PFCD is the timing of discontinuation of anti-TNF drugs. In our study, we found that fistulas can recur radiologically even among patients with prior radiological fistula healing. We can assume that even if radiological fistula healing is achieved, it is possible for fistulas to recur radiologically when appropriate IFX trough levels are not maintained. Therefore, while radiological fistula healing can be a treatment goal for the management of PFCD, but it should not be considered an indicator for discontinuation of IFX. Discontinuing anti-TNF treatment should be carefully decided between the physician and patient with PFCD, and this debatable issue should be discussed in future studies.

This study has several limitations. Due to its retrospective design, a significant number of patients were excluded from the analysis. A total of 172 patients diagnosed with PFCD were initially screened; however, more than half of the patients were excluded for various reasons. These major limitations regarding patient selection may have biased the results. Additionally, due to the small sample size and insufficient data for certain variables, it was difficult to obtain statistical significance in a few analyses. Moreover, our center's treatment protocol, in which medication is switched only in cases of severe disease worsening or severe adverse events despite low trough levels or ATI, may have also biased the results. However, the strengths of our study lie in that it is the largest study involving the pediatric population with PFCD assessing fistula response associated with IFX trough level. Moreover, we assessed the fistula response according to MRI findings, which is a reliable and objective predictor of long-term fistula healing.

## Conclusion

Higher serum IFX trough levels during induction and maintenance were associated with radiological fistula healing after 1-year of IFX treatment in pediatric patients with PFCD. Further prospective studies are warranted to provide a consensus for cut-off trough levels and the timing of IFX discontinuation, eventually improving the long-term outcomes of PFCD in pediatric patients.

## Data Availability

The raw data supporting the conclusions of this article will be made available by the authors, without undue reservation.
